# Supplementary material for: Carboxylic acid-modified metal oxide catalyst for selectivity-tunable aerobic ammoxidation
Source: Nat Commun. 2018 Mar 2;9:933. doi: 10.1038/s41467-018-03358-x (PMC5834450; doi:10.1038/s41467-018-03358-x)
Supplement: Supplementary file 1 — Supplementary Information [file 41467_2018_3358_MOESM1_ESM.pdf]

Carboxylic Acid-Modified Metal Oxide Catalyst for Selectivity-Tunable Aerobic Ammoxidation

Jia et al.

Supplementary Methods

Materials. All the chemicals used were of analytical grade and used as received unless otherwise stated. CH_3CN, pyridine, benzaldehyde, benzoic acid, phenol, NaAc, H_3PO_4, $KMnO_4$ and $MnAc_2$ were purchased from Tianjin Kermel Chemical Reagent Co. Ltd. HAc was purchased from Sinopharm Chemical Reagent Co., Ltd. Benzyl alcohol and terephthalonitrile were obtained from Aladdin Chemistry Co. Ltd. n-Hexanoic acid was purchased from TCI Shanghai. 4-Hydroxymethylbenzaldehyde and 4-cyanobenzyl alcohol were obtained from Innochem.

Supplementary Equations:

$$\text{Conversion} = \left(1 - \frac{\text{Moles of substrate}}{\text{Moles of substrate loaded initially}}\right) \times 100\% \qquad 1$$

$$\text{Selectivity of product} = \left(\frac{\text{Moles of product}}{\text{Moles of substrate converted}}\right) \times 100\% \qquad 2$$

Mass specific activity on benzyl alcohol oxidation

$$= \frac{\text{Moles of substrate converted}}{\text{Mass of catalyst} \times \text{Reaction time}} \qquad 3$$

Supplementary Figures

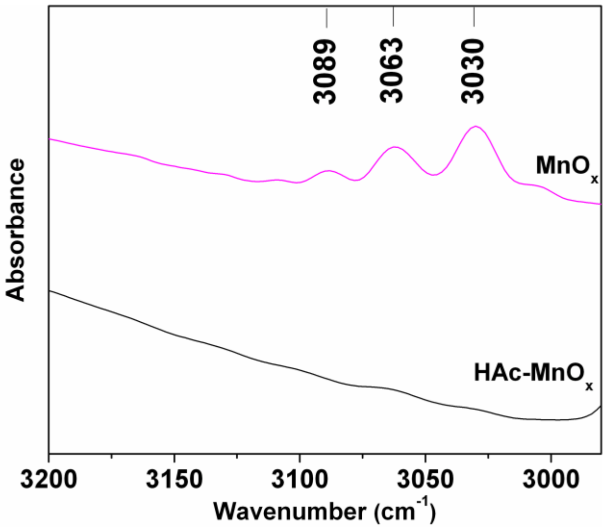

Supplementary Figure 1. FTIR spectra for the benzyl alcohol adsorption (2980 to 3200 cm^{-1}). The bands at 3030, 3063 and 3089 cm^{-1} are attributed to the C-H stretching vibrations of benzene ring in adsorbed benzyl alcohol.

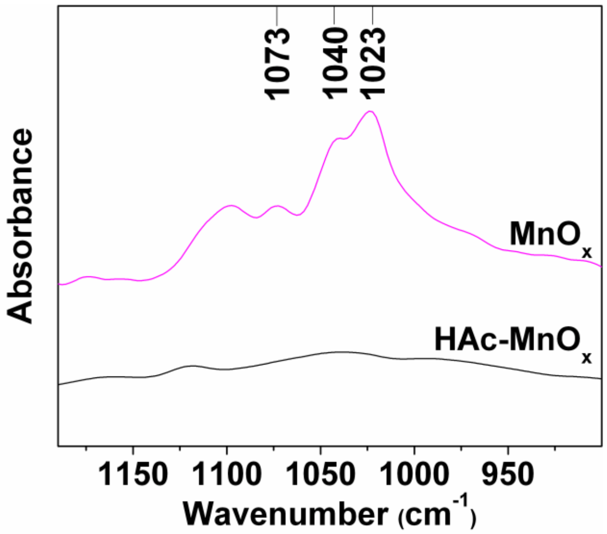

Supplementary Figure 2. FTIR spectra for the benzyl alcohol adsorption (900 to 1190 cm^{-1}). The bands at 1073, 1040 and 1023 cm^{-1} are attributed to the C-O stretching vibrations of metal-methoxy species in adsorbed benzyl alcohol.

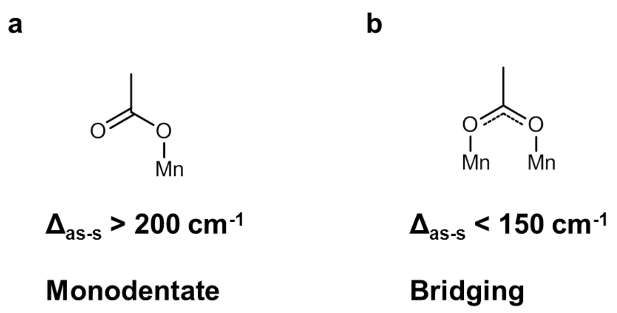

Supplementary Figure 3. Assignments of the different types of coordination modes (a-b) of the -COO group on the surface of MnO_x.[1] These assignments are based on the difference between symmetric and asymmetric stretching vibrations of -COO group.

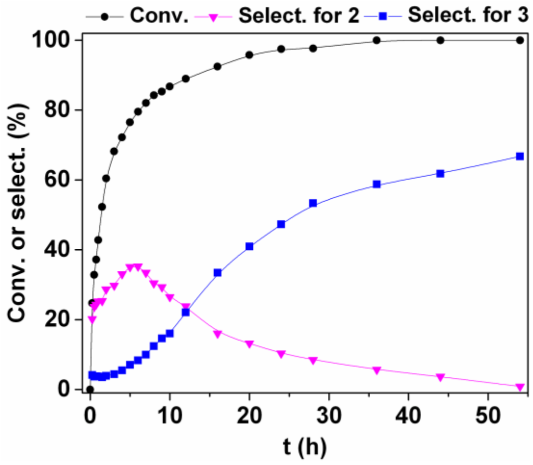

Supplementary Figure 4. Time course of aerobic ammoxidation of 4-hydroxymethylbenzaldehyde over MnO_x at 50 ^oC. Reaction conditions: 2.5 mmol 4-hydroxymethyl-benzaldehyde, MnO_x/substrate = 34 mol%, 25 mL CH_3CN, 0.3 MPa NH_3, 0.3 MPa O_2, 50 ^oC.

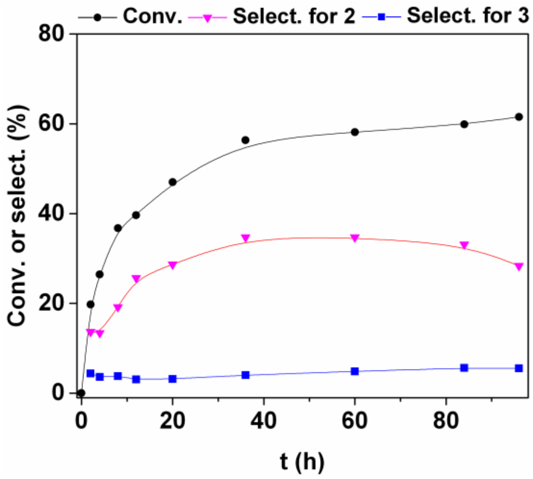

Supplementary Figure 5. Time course of aerobic ammoxidation of 4-hydroxymethylbenzaldehyde over MnO_x. Reaction conditions: 2.5 mmol 4-hydroxymethyl-benzaldehyde, MnO_x/substrate = 17 mol%, 25 mL CH_3CN, 0.3 MPa NH_3, 0.3 MPa O_2, 50 ^oC.

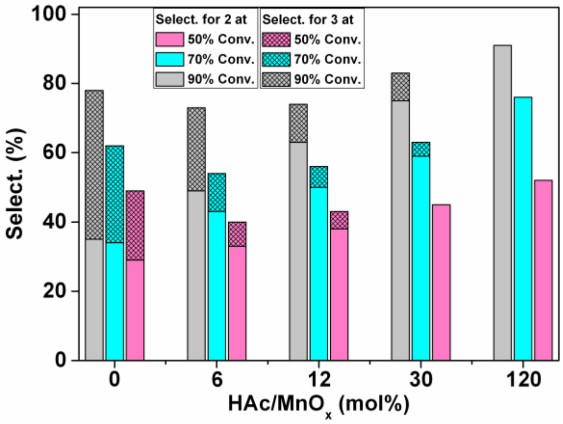

Supplementary Figure 6. Performance of MnO_x in ammoxidation of 4-hydroxymethylbenzaldehyde as a function of HAc/MnO_x. Reaction conditions: 2.5 mmol 4-hydroxymethyl-benzaldehyde, MnO_x/substrate = 34 mol%, 5 mL CH_3CN, 0.3 MPa NH_3, 0.3 MPa O_2, 80 ^oC.

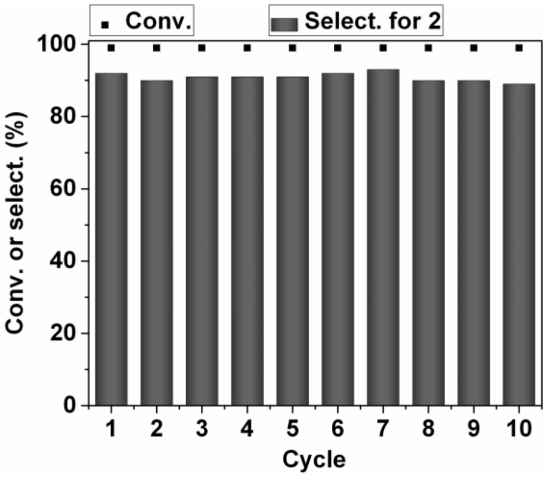

Supplementary Figure 7. Recyclability test results for selective ammoxidation of 4-hydroxymethylbenzaldehyde catalyzed by HAc modified MnO_x. Reaction conditions: 0.5 mmol 4-hydroxymethyl-benzaldehyde, 0.17 mmol MnO_x, HAc/MnO_x = 120 mol%, 5 mL CH_3CN, 0.3 MPa NH_3, 0.3 MPa O_2, 80 ^oC, 3 h. At the completion of the reaction, the solution was separated by centrifugation and analyzed by GC using the internal standard method. The catalyst was washed with CH_3CN 3 times and then regenerated by adding HAc (HAc/MnO_x = 120 mol%) to the reaction mixture for the cycle experiments.

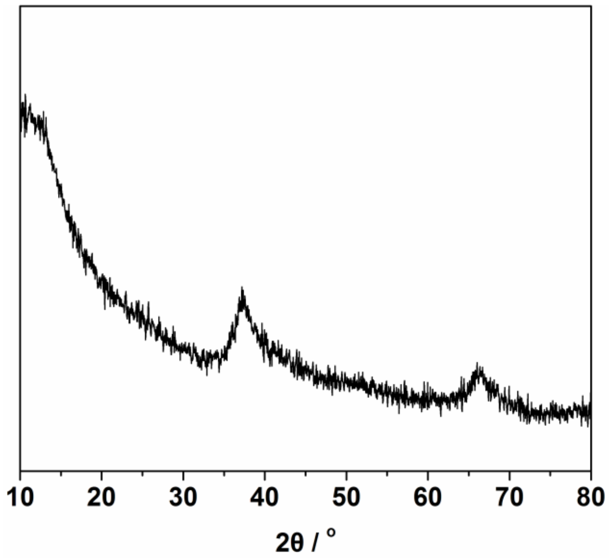

Supplementary Figure 8. XRD pattern of MnO_x.

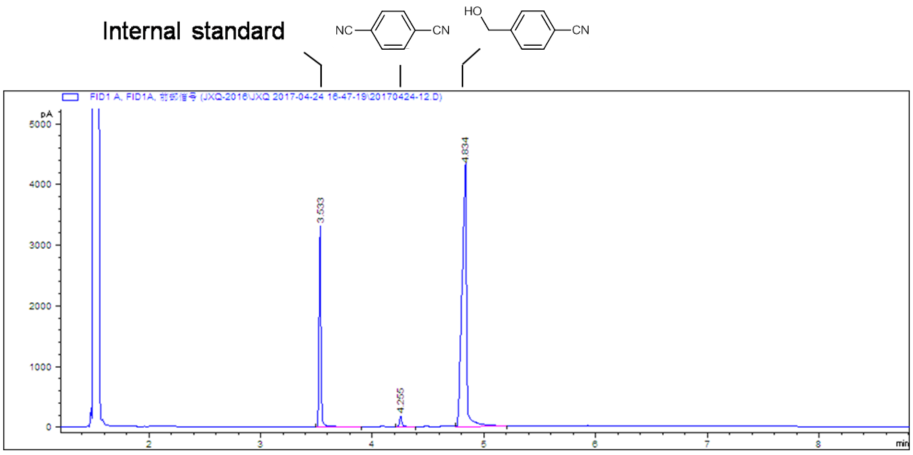

Supplementary Figure 9. GC spectrum of products from ammoxidation of 4-hydroxymethylbenzaldehyde (**Fig. 5** in the main text). Reaction conditions: 0.5 mmol 4-hydroxymethylbenzaldehyde, 0.17 mmol MnO_x, HAc/MnO_x = 120 mol%, 5 mL CH_3CN, 0.3 MPa NH_3, 0.3 MPa O_2. 80 ^oC, 3 h.

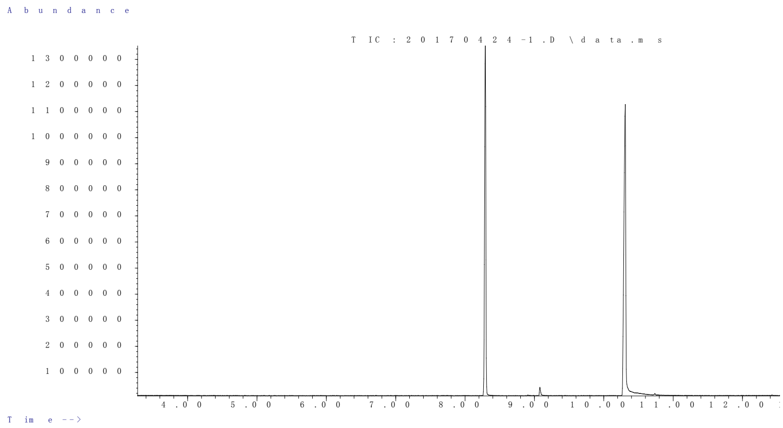

Supplementary Figure 10. GC part of GC-MS spectra of products from ammoxidation of 4-hydroxymethylbenzaldehyde (**Fig. 5** in the main text). Reaction conditions: 0.5 mmol 4-hydroxymethylbenzaldehyde, 0.17 mmol MnO_x, HAc/MnO_x = 120 mol%, 5 mL CH_3CN, 0.3 MPa NH_3, 0.3 MPa O_2. 80 ^oC, 3 h.

MS: m/z (%):133 (38) [M+], 132 (45), 104 (100), 77 (30)

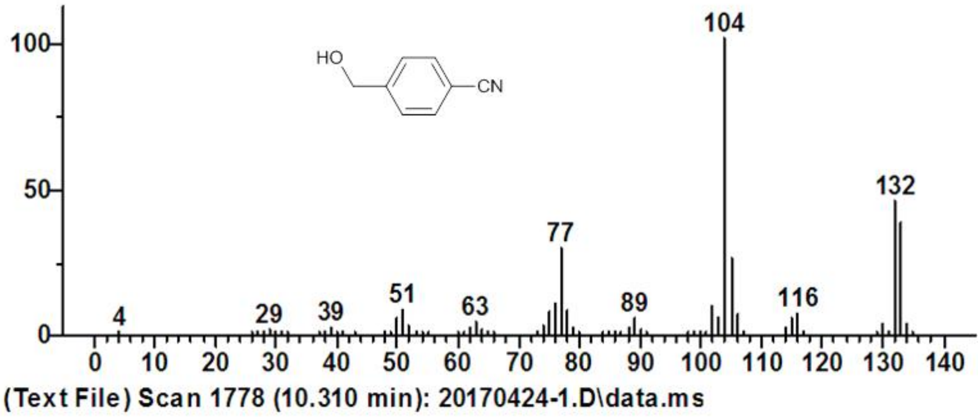

Supplementary Figure 11. Mass spectrum of **2**.

MS: m/z (%):128 (100) [M+], 101 (25), 75 (11), 50 (10) , 32 (14) , 28 (46)

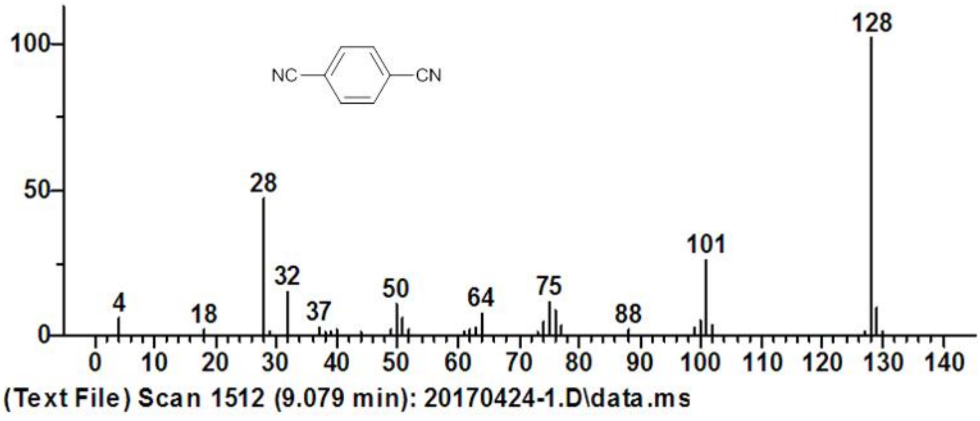

Supplementary Figure 12. Mass spectrum of **3**.

Supplementary References:

[1] Persson, P., Karlsson, M. & Öhman, L. O. Coordination of acetate to Al (III) in aqueous solution and at the water-aluminum hydroxide interface: A potentiometric and attenuated total reflectance FTIR study. *Geochim. Cosmochim. Acta* **62**, 3657-3668 (1998).
